# Do Great Minds Prefer Alike? Thirteen-Month-Old Infants Generalize Personal Preferences Across Objects of Like Kind but Not Across People

**DOI:** 10.3389/fpsyg.2018.02636

**Published:** 2018-12-18

**Authors:** Siying Liu, Renji Sun

**Affiliations:** ^1^School of Psychology and Cognitive Science, East China Normal University, Shanghai, China; ^2^School of Business, East China University of Political Science and Law, Shanghai, China

**Keywords:** preference, generalization, infant cognition, infants, visual habituation

## Abstract

Human preferences are person specific because different individuals do not necessarily have the same preference. Although existing empirical evidence demonstrates that infants have a basic understanding about people’s preferences, there remained one question as to whether infants appreciate that a person’s preference can be generalized to objects that belong to the same kind. This study addressed this gap with 13-month-old Chinese infants. In Experiment 1, infants were first habituated to an actor preferring a target object over a different shaped distractor object. Next, the objects’ position and colors were changed and infants watched the actor from habituation and a new actor each alternated preference between the two objects. Results revealed that infants looked longer at the event when the old actor preferred the distractor object. Throughout the experiment, infants were also presented with additional tests in which each actor requested them to visually identify his or her preferred object. Results showed that infants identified the different colored target object for the old actor but not for the new actor. Experiment 2 removed the additional tests and replicated the results of Experiment 1. Experiment 3 confirmed that 13-month olds could differentiate the two different colored target objects. Together, the present findings provide the first known evidence that 13-month olds expect object preferences to be generalized across objects of like kind but not necessarily across individuals. Such sensitivity to the rules guiding preference generalization could help infants predict people’ behaviors and facilitate more successful social interactions.

## Introduction

Even in the early years of development, children are often confronted with a variety of human behaviors that they must learn to correctly make sense of. One key piece of information they must be aware of is the distinction between conventional and non-conventional behaviors (Henderson and Woodward, 2012). Conventional behaviors are those that are shared between individuals within a particular community such as language and social conventions (e.g., bowing and waving) (Clark, 1992, 1993; Diesendruck and Markson, 2011). By contrast, non-conventional behaviors such as preferences and desires are person specific (Henderson et al., 2008; Henderson and Woodward, 2012). There is a growing body of research suggesting that infants can distinguish between these two types of behaviors and more specifically, they understand that personal preferences are non-conventional (Henderson and Graham, 2005; Buresh and Woodward, 2007; Henderson et al., 2008; Henderson and Woodward, 2012). The appreciation of the non-shared nature of personal preferences is key to the interpretation and prediction of ambiguous behaviors in novel contexts (Wellman, 1992; Knowles et al., 2001; Lieberman et al., 2005; Sommerville and Crane, 2009; Luo and Beck, 2010; Cannon and Woodward, 2012). For example, an infant might notice that one of his friends likes his toy car while another friend prefers to play with her doll. The understanding that each person’s preferences can be different means that the infant would not be surprised when his friends do not prefer the same object and more importantly, the infant can later use this knowledge to predict which toy each of his friend will choose in future instances. This ability to understand and predict people’s preferences in turn has fundamental importance to successful social interactions because it enables infants to treat people as unique individuals when it comes to desires and preferences (Buresh and Woodward, 2007).

There is no single comprehensive theory that can fully explain how infants acquire conceptual knowledge about human preferences. However, it has been proposed that since infants are dependent on their caregivers for the fulfillments of their personal desires, they spend a large proportion of their social interactions communicating their own desire and wants. In this process, infants encounter a large number of communicative failures when their desires are not well understood by others. These failures may play a crucial role in shaping infants’ understanding that people can have different preferences. Later as infants become more independent, they experience more incidents in which their desires are in conflict, or may be stopped by others. These incidents may again facilitate infants’ appreciation of the non-shared nature of personal preferences (Gopnik and Meltzoff, 1997). Another theory suggests that infants acquire knowledge about preferences through a simple process of observation. In their daily social encounters, infants have many opportunities to observe different people having different goals or preferences (e.g., mother prefers drinking tea while father prefers beer). These observations would in turn teach infants to associate preferences only to specific individuals (Buresh and Woodward, 2007).

The foundation for understanding preferences involves the appreciation that human behaviors are often goal or intention directed (Johnson, 2000; Sommerville et al., 2005; Biro and Hommel, 2007; Csibra and Gergely, 2007). Research evidence reveals that infants tend to interpret people’s actions such as gazes and reaches as reflections of their underlying intentions (Meltzoff, 1995; Woodward, 1998; Woodward and Sommerville, 2000; Woodward and Guajardo, 2002; Woodward, 2003; Behne et al., 2005; Sommerville et al., 2005; Johnson et al., 2007; Woodward et al., 2009; Luo and Baillargeon, 2010). Using a visual habituation paradigm, Woodward (1998) presented 5- to 9-month olds with the events in which an actor repeatedly reached and grasped one object (i.e., the target) but did not act on another object (i.e., the distractor). After infants habituated to the event, the objects changed color and position and infants were presented with two types of tests. In the Old Goal tests, the actor grasped the target that was in a new position. In the New Goal tests, the actor grasped the distractor that was in the position previously occupied by the target. It was found that infants demonstrated a novelty response (i.e., looked longer at an event) only toward the New Goal tests. These findings indicate that infants appreciate that the actor’s behaviors during habituation were driven by the goal of approaching and obtaining the target object; therefore they demonstrated a novelty response when the person suddenly changed his or her goal in the New Goal tests.

Studies using similar procedures further demonstrate that infants also understand that many goal-directed behaviors have an underlying disposition (such as preference for an object over another). The understanding of the disposition in turn helps infants to explain and predict a person’s goal-directed behaviors (Luo and Baillargeon, 2005; Luo and Johnson, 2009; Sommerville and Crane, 2009; Luo and Beck, 2010; Choi et al., 2018). In these studies, infants were assigned into either the Two Objects or One Object condition. In the Two Objects condition, infants habituated to a non-human agent approaching a target object in the presence of a distractor. They were then presented with the New Goal tests and Old Goal tests identical to those in the Woodward (1998) study. In the One Object condition, the procedure was the same as the Two Object condition except that when the agent approached the target object during habituation, the distractor was never present. Results showed that infants looked longer toward the New Goal tests only in the Two Objects condition and looking time was about equal for the two types of tests in the One Object condition (Luo and Baillargeon, 2005; Luo, 2011). It was reasoned that infants in the Two Objects condition seemed to interpret the agent’s goal directed behaviors as being driven by a disposition, namely a preference for the target object over the distractor because it justified why the agent repeatedly made a choice between the two objects. Infants also used this disposition to predict what the agent would later prefer, thus showing a novelty response when it preferred the distractor instead of the target. In the One Object condition, infants could understand that the agent’s goal to approach the target object. However, infants did not interpret this goal directed behavior as a preference because they were unable to predict what the agent’s goal would be when a new object (e.g., the distractor) was later introduced, thus showing equal looking time for the New Goal and Old Goal tests (Luo and Beck, 2010). These findings were further supported by studies using human agents. For example, infants interpreted a person repeatedly grasping one of two objects as an indication of her preference for a target object only if the person could detect both objects (Luo and Johnson, 2009). Together, these results demonstrate that infants tend to interpret goal directed behaviors toward one of two objects as a preference for one object over the other.

The next step toward more sophisticated understanding of personal preferences concerns the knowledge that different individuals may not share the same preference (Repacholi and Gopnik, 1997; Graham et al., 2006; Buresh and Woodward, 2007; Henderson and Woodward, 2012; Novack et al., 2013). In a study by Buresh and Woodward (2007), 13-month-old infants repeatedly watched an actor grasped one of two objects and expressed his or her preference for it (e.g., picking up the object and saying “oh, hmmm” in a positive tone). After habituation, half of the infants watched the same actor from habituation alternated his or her preference for either the same object from habituation or the other object (i.e., the Single Actor condition). Another half watched a new actor alternated his or her preference between the two objects (i.e., the Switch Actor condition). Results showed that infants in the Single Actor condition looked longer when the actor changed his or her preference. However, those in the Switch Actor condition did not show the same novelty response. Therefore, infants understand that different individuals do not necessarily share the same preference for an object. Other studies with younger infants have also yielded similar findings (Buresh and Woodward, 2007; Henderson and Woodward, 2012; Novack et al., 2013).

Although infants have been shown to recognize that one person’s preference does not necessarily generalize across people, other studies indicate that infants would generalize preference across different objects. After observing an adult expressing happiness to one type of food and disgust to another, 18-month olds successfully predicted that the adult would later prefer food associated with her prior positive emotions (Repacholi and Gopnik, 1997). In a more recent study, Luo and Beck (2010) familiarized 16-month olds with the events in which an actor faced two objects of different colors (i.e., a red toy pepper and a black cup, then a red pyramid, and a yellow toy house) and repeatedly pointed to the red objects, indicating his or her preference for red. In a subsequent test, the actor pointed to either a red front screen or a green front screen. Results revealed that infants expected the actor to continue preferring a red screen, thus generalized the actor’s color preference to a new object. Based on these past findings, infants are shown to be able to generalize a person’s preference to a different object according to the emotions associated with the objects or the color of the objects.

Taken together, past research indicates that infants appreciate the goal-directed nature of human behaviors and understand that some of these behaviors can reflect people’s preferences (Repacholi and Gopnik, 1997; Woodward, 1998; Buresh and Woodward, 2007; Biro and Hommel, 2007; Luo and Johnson, 2009; Luo, 2011). They also understand that one person’s preference does not necessarily generalize to another individual but can be generalized to other objects (Buresh and Woodward, 2007; Luo and Beck, 2010; Novack et al., 2013). However, there is another instance that a person’s preference can often be generalized. For example, a young infant might notice that his friend’s preference for cars could generalize to toys of like kind (i.e., toy cars of all colors or types). To date, no studies had explored whether infants would generalize a person’s preference to another object of like kind (i.e., an object that maintained the shape of the previous object but is in a different color). Furthermore, no studies had examined whether infants would expect the generalization of object preference to be person specific.

In order to investigate whether infants understand that personal preferences can be generalized to objects of like kind but not necessarily to other individuals, the present study adopted an experimental method that incorporated the visual habituation paradigm and the intermodal preferential looking (IPL) paradigm. This is because in past studies that utilized the visual habituation paradigm, novelty responses toward a test event (e.g., looking longer at an event that disrupted a previously established behavior) was used as the main indicator of infants’ understanding of personal preference. However, infants’ novelty response does not provide a full account of infants’ mental processes when looking at an event (Oakes, 2010). Thus, the present study also employed the IPL paradigm which involved infants being verbally presented with previously taught object-related information and are then required to identify the target object by looking at the objects on screen (Golinkoff et al., 2013). This paradigm could obtain more detailed information about infants’ looking responses such as the exact location and the duration of specific fixations when they watch a person’s goal-directed behaviors (Golinkoff et al., 2013).

In Experiment 1, 13-month-old infants were habituated to an actor explicitly expressing his or her preference for a target object while ignoring a different shaped distractor object. After habituation, the objects changed color (i.e., the test target and test distractor) and were switched in position. Infants were then tested as they watched the actor from habituation (i.e., the habituation actor) and a new actor preferring either the test target or the test distractor. It was hypothesized that if 13-month-old infants understood that a person’s preference could be generalized across objects of like kind, they would look longer when the habituation actor preferred the test distractor. Second, if infants understood that the preference for one kind of object may not be generalized to another individual who had not explicitly expressed his or her preference before, they would look equally long when the new actor preferred the test target or the test distractor.

Moreover, tests known as the “which is it” tests with features derived from the IPL paradigm were arranged throughout the present study. In these tests, the test target and test distractor were presented to the infants and the habituation actor and the new actor each asked the infants to find his or her preferred object. These tests investigated whether infants could actively identify a person’s preference thus provide more direct indications of their understanding about the generalizability of personal preferences. If 13-month olds had an understanding that personal preferences can be generalized across objects of like kind for the same individual but not necessarily for other people, they would look at the test target more than they would at the test distractor only for the habituation actor.

Experiments 2 and 3 were conducted as control experiments. In Experiment 2, the “which is it” tests were removed from the study to explore whether the results would replicate those in Experiment 1. In Experiment 3, infants underwent the same habituation process from Experiment 1. However, infants were then presented with “which is it” tests during which the habituation actor requested them to identify his or her preferred object between the two different colored target objects. This experiment investigated whether infants could perceive the two targets as different objects and recognize the habituation actor’s specific preference.

## Experiment 1

### Materials and Methods

#### Participants

Thirty full-term infants (*M*_age_ = 13 months, 8 days; range = 12 months, 5 days–14 months, 20 days; 14 males) were recruited from a public advertisement released in Shanghai. All infants were selected based on monolingual exposure to Mandarin Chinese. Five additional infants were tested but excluded from the final sample because of failure to complete the procedure due to distress (*n* = 2) and insufficient eye-tracking data (*n* = 3).

#### Stimuli

**FIGURE 1:**
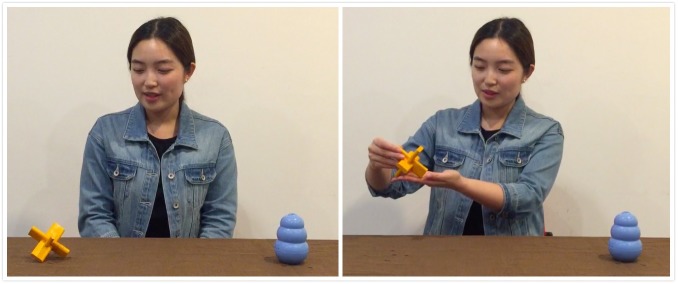
An example of habituation trial used in all experiments. The habituation actor gazed at and expressed her preference for the target object. The individual who appears here gave signed written consent for her image to be published in this article.

**FIGURE 2:**
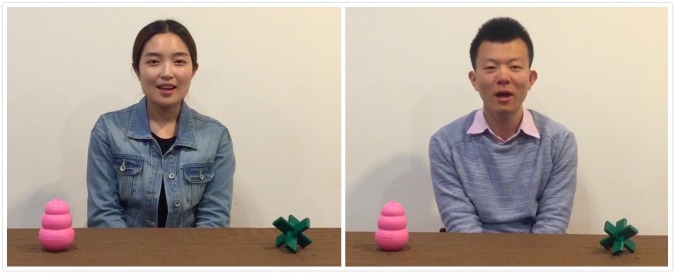
An example of “which is it” test pair used in Experiment 1. The two objects from habituation changed color and position. The habituation actor asked participants to looked at her preferred object, and this action was then repeated by a new actor. The individuals who appear here gave signed written consent for their images to be published in this article.

Four novel objects (two pairs of objects, each pair contains objects that are same in shape but differ in color) were used (see Figures 1, 2). The present studies used object pairs that differ in shape because past studies found that infants tend to use an object’s shape as an indicator of object kind when making generalizations. This could be because an object’s shape is a more stable than other features such as color and size (Graham and Poulin-dubois, 1999; Xu et al., 2004; Yoon et al., 2008).

#### Ethics Statement

The present study had been ethically reviewed and received ethics approval from the East China Normal University Committee on Human Research Protection (HR 073-2018). Infants’ parents gave written informed consent in accordance with the Declaration of Helsinki.

#### Procedure

The experiment consisted of a habituation phase and a post-habituation test phase. In the habituation phase, infants sat on their caregivers’ lap approximately 60 cm from a Tobbi T120 eye tracker in a curtained room where infants could only see the eye tracker screen. The eye tracker was connected to a computer (controlled by the experimenter) that presented video stimuli on the eye-tracker screen using the TobiiStudio software. A video camera was placed above the eye tracker to record infants’ gazes and the recordings were relayed to another room in which a coder (who was blind to the trials that had been run) coded infants’ looking time and calculated the habituation criteria online using the computer program jHab (Casstevens, 2007).

At the start of the habituation phase, infants first saw an Actor Familiarization video. In the video, two Chinese actors (one male and one female) played a familiarization game. Each actor appeared from behind a table alone saying “*wo zai zhe li*” “here I am” in Mandarin and then the two appeared together side by side, saying “*kan, wo men dou zai zhe li*” “Look, we are all here.” This video served to familiarize infants with the two actors. Infants then watched a habituation trial. In this trial, one of the actors (i.e., the habituation actor) appeared on screen with two novel objects (one from each object pair) in front of him or her side by side on a table. He or she first attracted infants’ attention by making direct eye contact and saying “*ni hao*” “hi.” The actor then gazed at one of the two objects (i.e., the target), smiled and said “*wo xi huan zhe ge*” “I like this.” Next, the actor grasped the target and indicated his or her preference again by saying “*wo xi huan zhe ge dong xi*” “I like this object.” After that the actor maintained his/her final position (see Figure 1). The other object (i.e., the distracter) was acted upon. Looking time for the habituation trial was started when infants first looked at the trial on screen, and the trial was ended when infants looked away from the screen for more than two consecutive seconds or after 120 s had elapsed. Infants were presented with the same habituation trial repeatedly until their total looking on three consecutive trials fell below half of the total amount of time they looked on the first three trials or until 15 trials had elapsed (indicating that the habituation criteria had been reached).

Throughout the habituation phase, infants were also presented with tests named the “which is it” tests. There were three test pairs (six test trials) in total. In the “which is it” tests, the two objects from the habituation phase were replaced with different colored ones (i.e., the test target and test distractor) and their sides were reversed. In the first trial of each test pair, the habituation actor first appeared and asked the infants “*Nihao, wo xi huan na yi ge? Ni kan jian wo xi huan de dong xi le ma*” “Hi, which one do I like? Can you find the object I like?” He or she then put his or her head down and maintained this final position. This was followed by the second trial of the pair during which infants saw a new actor who was present in the Actor Familiarization video but not in the habituation phase asked infants the same questions (see Figure 2). Each “which is it” test pair was presented after the passage of five habituation trials (e.g., the first pair of “which is it” tests was presented after the fifth habituation trial; the second pair began after the 10th habituation trial and the third pair began after the fifteenth habituation trial). If the habituation criteria were reached but infants had not finished watching all three “which is it” test pairs, they were then shown the remaining test pairs together immediately after infants had reached the habituation criteria. The “which is it” test trials were coded the same way as the habituation trials, starting when infants first looked at the screen and stopped when they looked away from the screen for more than two consecutive seconds or after 120 s had passed. Infants’ looking pattern (e.g., fixations) toward the objects in the “which is it” tests were recorded directly by the eye tracker.

**FIGURE 3:**
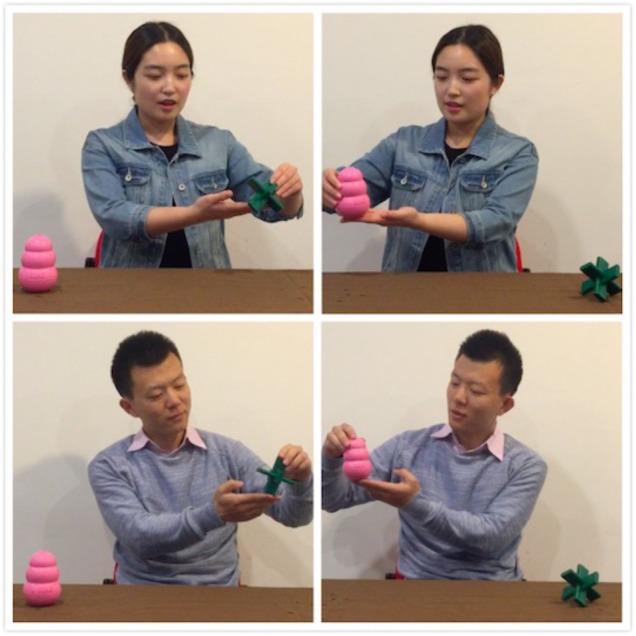
An example of post-habituation test pair 1 (top) and 2 (bottom) used in Experiments 1 and 2. After habituation, the habituation actor first alternated preference between the different colored target (target test trial on top left) and distractor (distractor test trial on top right). The new actor then performed the same actions. The individuals who appear here gave signed written consent for their images to be published in this article.

Following the habituation phase, infants underwent the post-habituation phase during which they were presented with four post-habituation test pairs (eight trials in total). In test pair 1, infants watched the habituation actor alternated his or her preference between the target (i.e., the target test trial) and the distractor (i.e., the distractor test trial). In test pair 2, infants watched the new actor presented the target test trial and distractor test trial (see Figure 3). These two test pairs were then repeated (test pair 3 was the same as test pair 1; test pair 4 was same as test pair 2). The actors’ action sequences and online coding procedure (including how these trials were started and ended) were identical to those in the habituation phase. At the end of the post-habituation test phase, infants watched a final pair of “which is it” tests.

The following factors were counterbalanced as much as possible across infants: the target object, the first test trials (i.e., half of the infants watched the actors presented the target test trial first and another half watched the actors presented the distractor test trial during each test pair in the post-habituation phase), and the gender of the actors who presented the habituation trials and post-habituation test trials (i.e., infants who watched the male actor during habituation watched the female at the post-habituation test phase and *vice versa*).

### Results

#### Preliminary Analyses

Infants habituated in an average of 8.97 trials (*SD* = 2.44). To test if infants’ looking time during the post-habituation phase was affected by the timing of the test pair, the test trial type and the first test trial type, a 4 (test pair: first, second, third, and fourth) × 2 (test trial type: target, distracter) × 2 (first test trial: target, distracter) mixed-design analysis of variance (ANOVA) with test pair and test trial type as the within-subject factors was conducted on infants’ average looking time toward for the post-habituation test trials. The ANOVA revealed a significant main effect of test pair, *F*(3, 28) = 43.15, *p* < 0.001, ${\text{η}}_{\text{Partial}}^{2}$ = 0.60. To further explore this main effect, paired-samples *t*-tests were conducted on infants’ total looking time for each test pair. Results showed that infants looked significantly longer toward test pair 1 (*M* = 29.83, *SD* = 10.94) than they did toward test pair 2 (*M* = 19.07, *SD* = 9.04), *t*(29) = 5.26, *p* < 0.001, *d* = 1.07, and *r* = 0.47, test pair 3 (*M* = 13.23, *SD* = 7.86), *t*(29) = 7.31, *p* < 0.001, *d* = 1.74, and *r* = 0.66, and test pair 4 (*M* = 9.22, *SD* = 5.76), *t*(29) = 9.47, *p* < 0.001, *d* = 2.36, and *r* = 0.76. They also looked significantly longer toward test pair 2 than 3, *t*(29) = 3.84, *p* < 0.001, *d* = 0.69, and *r* = 0.33 and 4, *t*(29) = 5.45, *p* < 0.05, *d* = 1.30, and *r* = 0.55. Looking time for test pair 3 was also significantly longer than test pair 4, *t*(29) = 2.78, *p* < 0.05, *d* = 0.58, and *r* = 0.28. Therefore, there was a decline of attention across test pairs during the post-habituation test phase and this result could be justified by the finding that infants’ attention tend to be the highest when they were first presented with an event and decline after repeated presentation (Henderson et al., 2008). The mixed-design ANOVA also yielded a significant main effect of test trial type, *F*(1, 28) = 39.90, *p* < 0.05, and ${\text{η}}_{\text{Partial}}^{2}$ = 0.59. This main effect was qualified by a significant two-way interaction between test pair and test trial type, *F*(3, 28) = 21.01, *p* = 0.02, and ${\text{η}}_{\text{Partial}}^{2}$ = 0.43 (explained in the main analyses). No other effects reached significance.

#### Main Analyses

##### Test trial type analyses

To further explore the significant two-way interaction between test pair and test trial type, a paired samples *t*-test was conducted on infants’ looking to the target and distracter test trials for the four test pairs. In test pair 1, infants looked significantly longer toward the distractor test trial (*M* = 19.05, *SD* = 7.54) than they did toward the target test trial (*M* = 10.78, *SD* = 5.48), *t*(29) = 6.15, *p* < 0.001, *d* = 1.25, and *r* = 0.53. They also looked significantly longer toward the distractor test trial (*M* = 8.82, *SD* = 5.81) than the target test trial (*M* = 4.41, *SD* = 2.71) in test pair 3, *t*(29) = 5.34, *p* < 0.001, *d* = 0.97, and *r* = 0.44. In test pair 2, there was no significant difference between looking time for the target (*M* = 9.71, *SD* = 5.01) and distractor test trial (*M* = 9.36, *SD* = 4.71), *p* > 0.05. In test pair 4, looking time was also not significantly different between the target (*M* = 4.93, *SD* = 3.61) and distractor test trial (*M* = 4.27, *SD* = 2.49), *p* > 0.05. These results indicate that in test pair 1 and 3 (which were presented by the habituation actor), infants showed longer looking each time the actor preferred the distractor instead of the target. For test pair 2 and 4 (which were presented by the new actor), infants did not discriminate between the target and distractor test trial.

**FIGURE 4:**
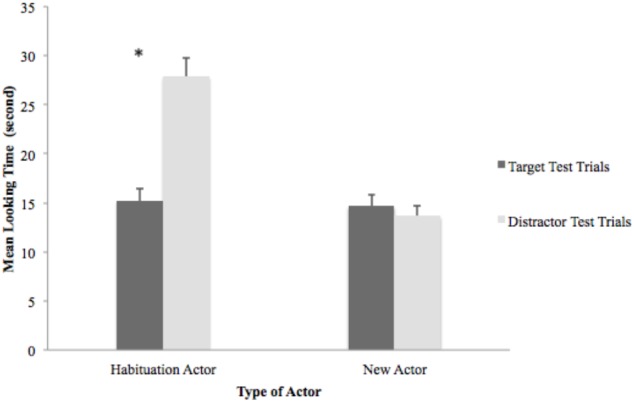
Average target and distractor looking time for the habituation and new actor during the post-habituation tests.

In order to explore infants’ differential looking response across all test pairs, a paired samples *t*-test was last conducted on infants’ mean total looking time for each type of test trials and for different actors. Results revealed that for the habituation actor, infants looked significantly longer toward the distractor test trials (*M* = 27.87, *SD* = 10.18) than they did toward the target test trials (*M* = 15.18, *SD* = 6.64), *t*(29) = 7.46, *p* < 0.001, *d* = 1.48, and *r* = 0.59. There was no significant difference in looking time between the target test trials (*M* = 14.65, *SD* = 6.25) and the distractor test trials (*M* = 13.65, *SD* = 5.91) for the new actor, *p* > 0.05 (see Figure 4).

##### “Which Is it” tests analyses

To investigate infants’ looking pattern when they were asked to find the object the actors preferred, four main variables that measured infants’ looking time toward the test target and test distractor were analyzed below. The mean looking time for each of these variables was shown in Table 1.

**Table 1:** Mean looking time and counts on the target and distractor for habitation actor and new actor in “Which Is It” tests (with standard deviations in parentheses).

	Habituation actor		New actor	
	Target	Distractor	Target	Distractor
Time to first fixation (s)	35.08 (8.49)	32.08 (17.53)	27.35 (12.20)	32.13 (13.29)
First fixation duration (s)	0.85 (0.45)	0.54 (0.39)^∗^	0.50 (0.26)	0.74 (0.37)^∗^
Total fixation duration (s)	6.38 (3.39)	1.21 (1.22)^∗^	1.95 (1.86)	2.97 (2.09)
Fixation count (times)	28.37 (15.09)	5.30 (4.77)^∗^	8.53 (7.91)	12.43 (7.49)

*^∗^Different from the target, p < 0.05.*

###### Time to First Fixation

This variable measured the time from which each actor first asked the question “which one do I like” until the infants first fixated on each of the object. A chi-square analysis was first conducted on the number of infants who looked at the test target first. For the habituation actor, there was no significant difference in the number of infants who looked that the target first (*M* = 14) and the distractor first (*M* = 16), *p* > 0.05. For the new actor, the number of infants who looked at target first (*M* = 12) was not significantly different from the number of infants who looked at the distractor first (*M* = 18), *p* > 0.05. A paired samples *t*-test was then conducted on the average time to first fixation for infants who looked at both objects. Results showed that there was no significant difference in the average time to first fixation for both actors, *p* > 0.05. Therefore, infants chose to first look at the test target and distractor equally after being asked to identify the actors’ preferred object.

###### First Fixation Duration

A paired samples *t*-test was then performed on infants’ average first fixation duration. For the habituation actor, the *t*-test revealed that infants’ first fixation was significantly longer for the test target than for the distractor, *t*(29) = 3.49, *p* = 0.002, *d* = 0.74, and *r* = 0.35. For the new actor, first fixation was significantly longer for the test distractor than for the target, *t*(29) = 3.91, *p* = 0.001, *d* = 0.76, and *r* = 0.36. Therefore, infants looked longer at the test target during their first fixation only for the habituation actor.

###### Total Fixation Duration

To test the total time infants fixated on each of the objects, a paired samples *t*-test was conducted on the average total fixation duration. For the habituation actor, infants’ total fixation duration was significantly longer for the test target than for the distractor, *t*(29) = 7.79, *p* < 0.001, *d* = 2.03, and *r* = 0.71. For the new actor, infants total fixation duration was not significantly different for the test target and the distractor, *p* > 0.05. Therefore, when asked by the habituation actor to find the object he or she preferred, infants spent longer fixating on the test target than they did on the distractor and this pattern was not observed for the new actor.

###### Total Fixation Count

A paired samples *t*-test was also performed on total fixation count that measured the total number of fixations infants had for each object. For the habituation actor, infants fixated on the test target significantly more than they did on the distractor, *t*(29) = 7.89, *p* < 0.001, *d* = 2.06, and *r* = 0.72. As for the new actor, the number of fixations was not significantly different for the test target than for the distractor, *p* > 0.05. Thus, infants fixated on the test target more frequently when asked by the habituation actor to identify his or her preferred object.

### Discussion

The findings from Experiment 1 provide some insights into infants’ understanding of the generalizability of human preferences. First, after repeatedly watching the habituation actor indicating his or her preference for the target object while ignoring the distractor object, 13-month olds looked longer only when the actor later preferred the different shaped test distractor but not the different colored test target. Therefore, infants seem to recognize that when a person prefers a particular object, he or she may also prefer another object of the same shape but different color. Moreover, it was found that infants’ looking time toward the test target and distractor trials did not differ for a new actor who never expressed his or her preference for any of the objects before. Thus, infants found the events equally acceptable when the new actor preferred the test target or distractor. This important finding further suggests that infants do not expect the preference for objects of a particular kind (i.e., objects that share the same shape but only differ in color) to generalize to another individual whose preference is unknown.

Furthermore, key findings from the “which is it” tests provide incremental evidence about infants ability to generalize preferences. When asked by the habituation actor to look at his or her preferred object, 13-month olds looked longer at the test target during their first fixation, fixated on the test target more times, and also for a longer period of time. Therefore, infants in the present study did not only show a novelty response when the habituation actor changed his or her previous preference but also actively identified the actor’s preferred object even if the object had changed color. By contrast, when the new actor asked the infants to find his or her preferred object, 13-month olds did not discriminate between the test target and distractor. This again illustrates that although infants expect the same person to prefer objects that only differ in color, they do not hold the same expectation for another individual who has never shown his or her preference before.

It is important to note that out of the four variables that measured infants’ fixations in the “which is it” tests; infants’ average time to first fixation did not differ for the test target and distractor. This reveals that infants chose to first look at the target and distractor equally after the actors requested them to visually locate their preferred object. One possible explanation is that during the “which is it” trials, the objects were switched in position and in different colors; thus, infants could have spent the first look checking where the objects were or how the objects’ appearance had changed. This could in turn result in infants randomly looking at the two objects for their first fixation. Nonetheless, the other three variables indicate that overall infants in Experiment 1 fixated at the test target more and longer for the habituation actor. This demonstrates that infants possess at least a basic understanding about how a person’s object preference can be generalized.

Since infants in Experiment 1 were presented with two types of tests, it was possible that infants’ performance in the post-habituation tests was affected by the prior “which is it” tests. That is, when the habituation actor directly asked infants to find his or her preferred object in the “which is it” tests during the habituation phase, the questions such as “which one do I like? Can you find the object I like” could have implied that one of the two test objects in front of the actor must be what the he or she preferred. Later in the post-habituation tests, infants could have assumed that the different colored test target was the correct choice for the habituation actor using their ability to categorize objects by shape. In order to investigate whether the “which is it” tests had such a priming effect on infants’ responses in the subsequent post-habituation tests, Experiment 2 was conducted as a control condition in which all the “which is it” tests were removed from the procedure.

## Experiment 2

### Materials and Methods

#### Participants

Thirty full-term infants (*M*_age_ = 13 months, 25 days; range = 13 months, 12 days–14 months, seven males) were recruited from four community early education centers in Shanghai. All infants were selected based on monolingual exposure to Mandarin Chinese. Four additional infants were tested but excluded from the final sample because of failure to complete the procedure due to distress (*n* = 1) and insufficient eye-tracking data (*n* = 3).

#### Procedure

The stimuli and procedure were similar to those in Experiment 1 with the following exceptions: first, all the “which is it” tests were removed from the procedure. Therefore, the procedure only consisted of the habituation trials followed by the post-habituation tests. Second, two object familiarization trials were presented in between the habituation phase and the post-habituation phase. In each of these trials, the test target and test distractor were presented on screen and were switched in position. There were no actors in these trials. The coding procedures (including how the trial was started and ended) in the object familiarization trials were the same as those in the habituation trials. These trials were used to familiarize infants with the test objects so their looking time in the post-habituation phase would not increase due to changes in the objects’ colors. Infants’ looking time during the post-habituation test trials were coded in the same manner as in Experiment 1; however, the coder was also asked to guess the type of trial (target vs. distractor test trial) that had been run. His guessing rate was about 46.67% correct.

### Results

Infants habituated in an average of 7.13 trials (*SD* = 1.80). To test whether infants’ looking time in the post-habituation tests was affected by the timing of test pair and types of tests presented, a 4 (test pair: first, second, third and fourth) × 2 (test trial type: target, distracter) × 2 (first test trial: target, distracter) mixed-design ANOVA with test pair and test trial type as the within-subject factors was performed on infants’ average looking time toward for the post-habituation test trials. The ANOVA revealed a significant main effect of test trial type, *F*(1, 28) = 5.66, *p* < 0.05, and ${\text{η}}_{\text{Partial}}^{2}$ = 0.17. This suggests that infants’ looking time was different for the target and distractor test trials. No other effects reached significance.

To further explore the significant main effect of test trial type, a paired samples *t*-test was conducted on infants’ mean total looking toward the two types of test trials for different actors. For the habituation actor, infants looked significantly longer toward the distractor test trials (*M* = 35.50, *SD* = 13.56) than they did toward the target test trials (*M* = 29.16, *SD* = 9.88), *t*(29) = 2.33, *p* < 0.05, *d* = 0.53, and *r* = 0.26. There was no significant difference in looking time between the target (*M* = 27.61, *SD* = 11.06) and distractor test trials (*M* = 27.43, *SD* = 8.23) for the new actor, *p* > 0.05. Therefore, infants only looked longer toward the distractor test trials for the habituation actor.

### Discussion

Overall, key findings from Experiment 2 were consistent with those in Experiment 1. Using a simple visual habituation paradigm, infants who repeatedly watched the habituation actor preferring the target object later showed a novelty response only when the actor preferred the different shaped test distractor. Therefore, infants found it acceptable for the habituation actor to extend his or her preference to another object that maintained the shape of the original target. In addition, infants did not discriminate between the target and distractor test trials for the new actor. This was also in line with Experiment 1 and suggests that 13-month-old infants had some understanding that for a new actor who did not express his or her preference before, it is equally likely that she may prefer any of the two objects. Therefore, infants did not expect the new actor to maintain the habituation actor’s preference.

Since Experiment 2 was conducted without the “which is it” tests and the main results replicated those from Experiment 1, it is unlikely that the “which is it” tests in Experiment 1 had a priming effect on infants’ responses in the subsequent post-habituation tests. However, there remained another open question concerning whether 13-month olds in Experiments 1 and 2 differentiated the different colored targets (i.e., the target at habituation and the test target in the post-habituation tests). Past research demonstrates that by 11.5 months, infants can use color to individualize objects (Wilcox, 1999). However, since shape has been suggested to be a more fundamental cue for object individualization (Graham and Poulin-dubois, 1999; Xu et al., 2004), it was possible that infants in Experiments 1 and 2 prioritized shape over color and perceived the different colored targets as the same object. To examine this possibility, Experiment 3 included a habituation phase identical to those in Experiments 1 and 2; however, the post-habituation test phase consisted of only four “which is it” tests during which the habituation actor asked infants to find his or her preferred object between the target and the different colored test target. If infants perceived the different colored targets as two distinct objects, it was expected that they would look longer at the original target because it was the object that the habituation actor explicitly preferred during habituation. If Experiment 3 demonstrated that 13-month olds could encode both the shape and color of the objects and identify an individual’s specific preference, their performance in Experiments 1 and 2 could then be interpreted as the generalization of a person’s specific preference to a new object.

## Experiment 3

### Materials and Methods

#### Participants

Twenty-four full-term infants (*M*_age_ = 13 months, 22 days; range = 12–15 months, 6 days; 13 males) were recruited from four community early education centers in Shanghai. All infants were selected based on monolingual exposure to Mandarin Chinese. Six additional infants were tested but excluded from the final sample because of failure to complete the procedure due to distress (*n* = 3), insufficient eye-tracking data (*n* = 2), and technical failure (*n* = 1).

#### Procedure

The stimuli and habituation phase were identical to those in Experiment 2 with the following exceptions. First, in between the habituation phase and post-habituation test phase, infants watched two object familiarization trials. During each trial, infants saw the target from habituation and the different colored test target (the test target became the new distractor from this point on) side by side on a table and were reversed in position. These two trials were started and ended the same way as the habituation trials and they served to familiarize infants with the objects, especially the new distractor. Second, the post-habituation test phase only consisted of four “which is it” test trials. In each test trial, the habituation actor appeared on screen with the target and the new distractor in front of him or her. The actor then asked infants “*Nihao, wo xi huan na yi ge? Ni kan jian wo xi huan de dong xi le ma*” ‘Hi, which one do I like? Can you find the object I like?’ before putting his or her head down and maintained this final position. These four “which is it” trials were started and ended the same way as those in Experiment 1.

### Results and Discussion

Infants reached the habituation criteria in an average of 7.48 trials (*SD* = 2.10). Infants’ looking pattern during the “which is it” tests were analyzed using the following four variables:

#### Time to first fixation

A chi-square analysis was first conducted on the number of infants who looked at the test target first. Resulted indicated no significant difference in the number of infants who looked that the target first (*M* = 14) and the distractor first (*M* = 10), *p* > 0.05. A paired samples *t*-test was then conducted on the average time to first fixation for infants who looked at both objects. Results showed no significant difference between the average time to first fixation toward the target (*M* = 37.72, *SD* = 8.67) and the distractor (*M* = 38.46, *SD* = 15.18), *p* > 0.05. Therefore, infants did not discriminate between the target and distractor during their first fixation after the habituation actor asked them to find his or her preferred object. This could be because infants were checking the changes in the objects and thus looked at both objects at random for their first fixation.

#### First Fixation Duration

A paired samples *t*-test was conducted on infants’ average first fixation duration. It was found that infants’ first fixation was significantly longer for the target (*M* = 0.66, *SD* = 0.26) than for the distractor (*M* = 0.49, *SD* = 0.19), *t*(23) = 2.77, *p* < 0.05, *d* = 0.73, and *r* = 0.34.

#### Total Fixation Duration

To test the total time infants fixated on each of the objects, a paired samples *t*-test was conducted on the average total fixation duration. Infants’ total fixation duration was significantly longer for the target (*M* = 5.14, *SD* = 2.40) than for the distractor (*M* = 2.95, *SD* = 2.06), *t*(23) = 2.98, *p* < 0.05, *d* = 0.98, and *r* = 0.44.

#### Total Fixation Count

A paired samples *t*-test was last performed on total fixation count for each object. Infants fixated on the target (*M* = 21.04, *SD* = 6.05) for significantly more times than they did on the distractor (*M* = 15.58, *SD* = 5.91), *t*(23) = 3.06, *p* < 0.05, *d* = 0.91, and *r* = 0.42.

Analyses on the above variables reveal that after watching a person preferring one of two different shaped objects and later asked for his or her preferred object, 13-month olds fixated longer and more frequently on the original target object than they did toward another object that only differed from the original target in color. Because in the “which is it” tests, the actor clearly requested for the object she “liked” and there was only one target object for which the actor preferred in the habituation phase, the finding that infants identified the target object suggests that they are able to differentiate the two different colored objects and recognize the actor’s original preference. These findings were in line with those by Wilcox (1999) that show infants can use color to individualize two objects of the same shape. Therefore, it is highly likely that infants in Experiments 1 and 2 perceived the target and the different colored test target as two objects. Based on this finding, infants’ looking responses in Experiments 1 and 2 (i.e., longer looking time toward the distractor test trials for the habituation actor only; fixating longer and more frequently on the test target for the habituation actor in the “which is it” tests) were likely to reflect their ability to generalize one person’s specific object preference to a new object.

## General Discussion

The present study was designed to examine whether 13-month olds appreciate that a person’s object preference can be generalized to another object of the same shape but different color and whether infants expect this type of generalization to be person specific. In Experiment 1, infants repeatedly watched an actor preferring a target object while ignoring a distractor. Later when the target and distractor changed color and position, infants looked longer when the actor preferred the distractor. Moreover, when the actor asked infants to identify his or her preferred object in a series of “which is it” tests, infants fixated more and longer on the different colored target. By contrast, 13-month olds did not differentiate between the target and distractor for a new actor whose preference remained unknown throughout the experiment. These results demonstrate that infants expect a person to generalize his or her object preference to a different colored object but not to another individual. Experiment 2 removed the “which is it” tests and the key results of the visual habituation paradigm replicated those in Experiment 1. Experiment 3 provided evidence that 13-month olds could individualize the different colored target objects thus the results from Experiments 1 and 2 were likely to indicate infants’ ability to generalize a person’s specific preference to another object.

The above major findings support and extend previous literature in a number of ways. First, past studies have documented that infants are able to interpret goal-directed behaviors as preference for one object over others (Luo and Baillargeon, 2005; Luo and Johnson, 2009; Sommerville and Crane, 2009; Choi et al., 2018). In the present study, when faced with two objects, infants successfully identified the different colored target as the preferred object for the habituation actor, showing that they expected the actor to prefer one of the two objects. Second, past research indicates that infants are able to generalize people’s color preferences (Luo and Beck, 2010). The results of the present study further reveal that infants are also able to generalize a person’s object preference to another object of the same shape but different color. Third, infants have been shown to understand that the preference for a particular object is rather person specific (Graham et al., 2006; Henderson and Woodward, 2012). The present study adds to this finding by suggesting that infants also appreciate that the preference for a particular kind of objects is also person specific.

Methodologically, findings from the “which is it” tests provide additional evidence about infants’ understanding of human preferences. In past studies that used similar procedures, tests that involved an actor directly requesting a child to locate an object were adopted mainly to help infants familiarize with the actor’s appearance or changes in objects’ positions (Henderson and Woodward, 2012; Henderson and Scott, 2015). Thus, infants’ looking patterns during these tests were not closely examined. In the present study, the “which is it” tests reveal that infants did not only show a novelty response when an individual suddenly changed his or her preference but were also able to actively identify the object that the person prefers even if it had changed color. Thus, the results from the “which is it” tests add to the findings from the post-habituation tests to suggest that infants are able to generalize a person’s preferences to another object of the same shape but different color. Moreover, the finding that infants only actively identified the target object for the habituation actor further shows that they recognize that another individual might not necessarily share the same preference.

The paradigm adopted in the present study poses another possible explanation regarding why infants only identified the test target for the habituation actor but not for the new actor during the “which is it” tests in Experiments 1 and 2. That is, because the new actor was not present during habituation phase and later alternated his or her preference between the two objects during the post-habituation tests, infants could simply be confused about the new actor’s actions rather than fully understood that different individuals may not share the same preference. However, since six out of the eight “which is it” tests were arranged throughout the habituation phase that was before the new actor alternated his or her preference, infants in the present study had the opportunity to identify the new actor’s preferred objects before they could be confused by the his or her subsequent behaviors. Thus, the finding that infants did not looked longer and more frequently on the target for the new actor provides at least some evidence that infants are cautious when generalizing one person’s object preference to another individual.

The ability to generalize information across objects of like kind is important in all forms of social learning. This is because generalization saves infants the cognitive effort of repeatedly learning new information about an event or individual (Horváth et al., 2016). For example, after learning that a friend likes toy cars, an infant can then avoid having to update this information every time the friend prefers a toy car to other toys. Thus, the assumption that personal preferences can be generalized across objects of like kind helps infants to efficiently acquire knowledge regarding an individual’s likes and dislikes (Buresh and Woodward, 2007; Sommerville and Crane, 2009). It should be noted that the present study only used objects that differed in color. This is only one aspect that objects of the same kind can differ from each other. Thus, it is unknown whether infants would expect a person to generalize his or her preference for objects that differ in other aspects such as size and material or when the objects’ appearance differ in more than one aspect.

Previous works have also demonstrated that infants as young as 9-month olds understand that the preference for a particular object does not necessarily generalize across people (Henderson and Woodward, 2012). However, since the experimental tasks in the present study involved generalization across different colored objects, 13-month olds were selected due to this increase of task difficulty. Thus, it is unclear whether younger infants also have the same level of understanding about the generalizability of object preferences. Future studies should be conducted with younger infants to investigate the age that this understanding first emerges. This would in turn help shed insights into the role played by social experience in the understanding of preference. In addition, the present study only examined the ability to generalize object preference in Chinese infants. However, it has long been argued that infants’ learning process is constantly shaped and refined by culture (Tomasello, 1999, 2016). Since the Chinese culture (an collectivist culture) tends to emphasize more on collective interests while the Western culture (an individualistic culture) centralizes on the satisfaction of individual goals (Oyserman et al., 2002), there is a possibility that infants raised in Western countries could receive more training about individual needs and thus have more advanced understanding about human preferences. Therefore, findings from the present study should not be fully generalized to infants in Western countries prior to future replications.

In conclusion, the present study provides the first evidence that by 13 months of age, Chinese infants appreciate that an individual’s object preference can be generalized to another object of like kind. They also understand that different people may not necessarily share the same preference for an object kind. Building on findings from previous literature that suggest infants understand many of the basic rules guiding human preference, the current findings demonstrate that infants have a fairly sophisticated understanding of the exceptions and constraints embedded in human preferences. This form of understanding is likely to guide infants’ interpretation and predictions about people’s personal preferences during future social encounters.

## Data Availability

The raw data supporting the conclusions of this manuscript will be made available by the authors, without undue reservation, to any qualified researcher.

## Author Contributions

SL developed the study concept and created the study design. SL and RS conducted the experiment and data collection. SL performed the data analysis and interpretation and drafted the manuscript. All authors approved the final version of the manuscript for submission.

## Conflict of Interest Statement

The authors declare that the research was conducted in the absence of any commercial or financial relationships that could be construed as a potential conflict of interest.
